# Endovascular repair of abdominal aortic aneurysm associated with residual type B aortic dissection utilizing iliac branch endoprosthesis: a case report

**DOI:** 10.1186/s44215-025-00194-6

**Published:** 2025-02-07

**Authors:** Kunitaka Kumagai, Yuichiro Kishimoto, Takeshi Onohara, Rikuto Nii, Naoki Sumi, Nozomi Kishimoto, Yosuke Ikeda, Yuki Yoshikawa, Kazuma Yamane, Yasushi Yoshikawa

**Affiliations:** Department of Cardiovascular Surgery, Tottori University Hospital, 36-01, Nishicho, Yonago, Tottori, 683-8504 Japan

**Keywords:** Aortic dissection, Abdominal aortic aneurysm, Iliac branch endoprosthesis

## Abstract

**Background:**

Iliac branch endoprosthesis (IBE) can preserve the internal iliac artery. Endovascular aortic repair (EVAR) for abdominal aortic aneurysms associated with residual type B aortic dissection has been rarely reported, and IBE has not been utilized in any of the reported cases. Herein, we describe a rare case of EVAR using IBE for an abdominal aortic aneurysm and a common iliac artery aneurysm associated with residual type B aortic dissection after thoracoabdominal aortic replacement.

**Case presentation:**

A 70-year-old man underwent conservative treatment for acute type B aortic dissection 5 years ago. Contrast-enhanced computed tomography (CT) revealed type B aortic dissection and a thoracoabdominal aortic aneurysm, an abdominal aortic aneurysm, and an aneurysm in the right common iliac artery. Since the patient had a concomitant right common iliac artery aneurysm and reconstructing the right iliac artery would have been difficult in a one-stage operation, a two-stage surgical strategy for thoracoabdominal and abdominal aortic graft replacement, separately, was designed. Thoracoabdominal aortic graft replacement from the proximal descending aorta to the infrarenal abdominal aorta was performed using a triplex 24-mm prosthesis (Terumo Aortic, Inchinnan, UK) without any problem. We recommended abdominal aortic graft replacement; however, he preferred EVAR over abdominal aortic graft replacement. The risk of spinal cord ischemia was a concern because a conventional EVAR would require embolizing the right internal iliac artery. Therefore, EVAR was performed with IBE to preserve the right internal iliac artery. The postoperative course was uneventful, and no spinal cord injury was observed. Follow-up CT showed no enlargement of the aneurysms 1 year postoperatively.

**Conclusions:**

Despite anatomic limitations, EVAR for abdominal aortic and common iliac artery aneurysms associated with residual aortic dissection after thoracoabdominal aortic graft replacement can be safely performed by embolizing the branch to the false lumen and using IBE.

## Background

Iliac branch endoprosthesis (IBE) is a device that can preserve the internal iliac artery. Endovascular aortic repair (EVAR) for abdominal aortic aneurysms associated with residual type B aortic dissection has been rarely reported [1, 2], and IBE has not been utilized in any of the reported cases. Herein, we describe a rare case of EVAR using IBE for an abdominal aortic aneurysm and a common iliac artery aneurysm associated with residual type B aortic dissection following thoracoabdominal aortic replacement.

## Case presentation

**Fig. 1:**
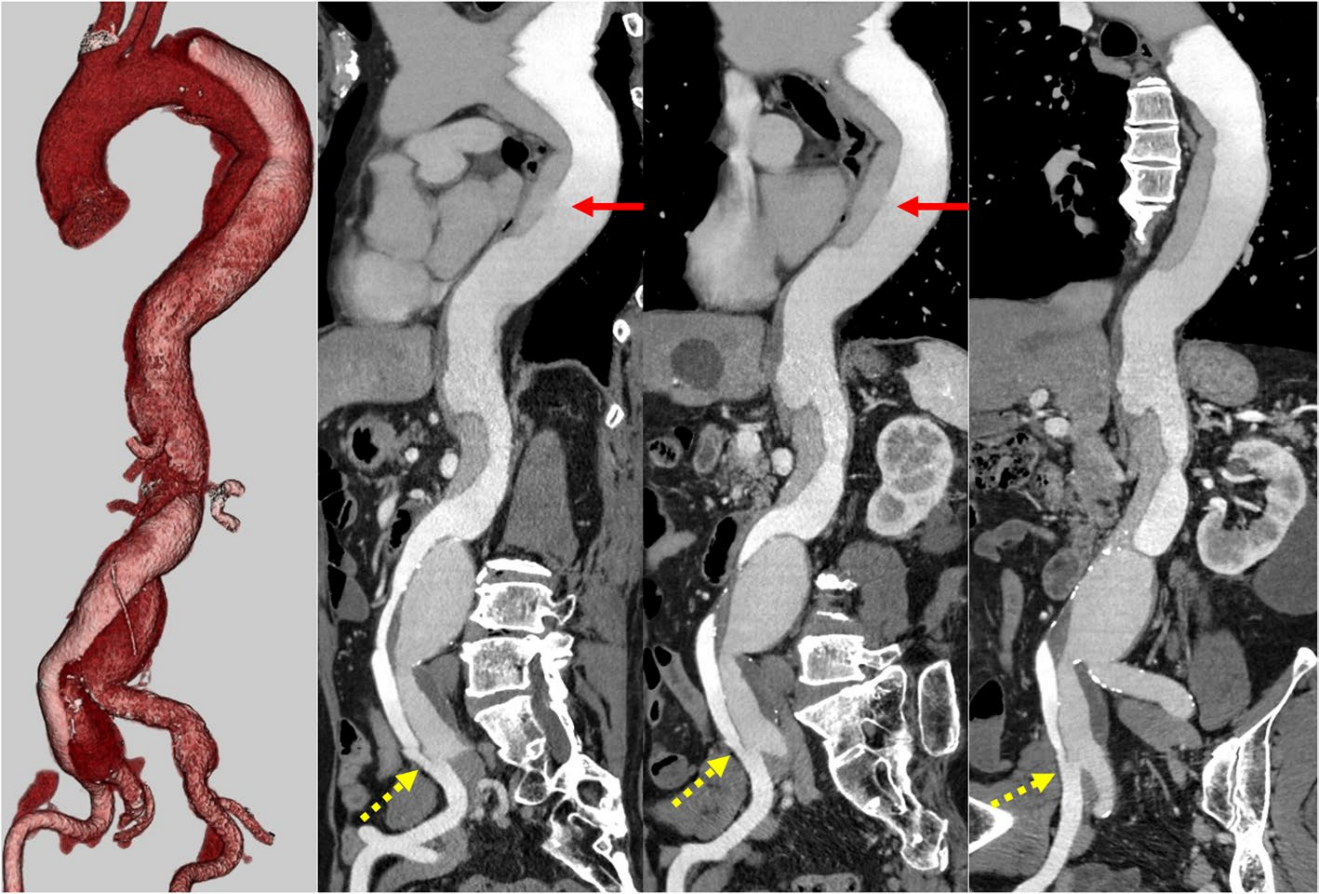
Preoperative contrast-enhanced CT (3-dimensional and stretch curved planar reconstruction). CT reveals type B aortic dissection and a thoracoabdominal aortic aneurysm, an abdominal aortic aneurysm, and a right common iliac artery aneurysm. Red arrows indicate the proximal tears. Yellow dotted arrows represent distal tears. CT, computed tomography

**Fig. 2:**
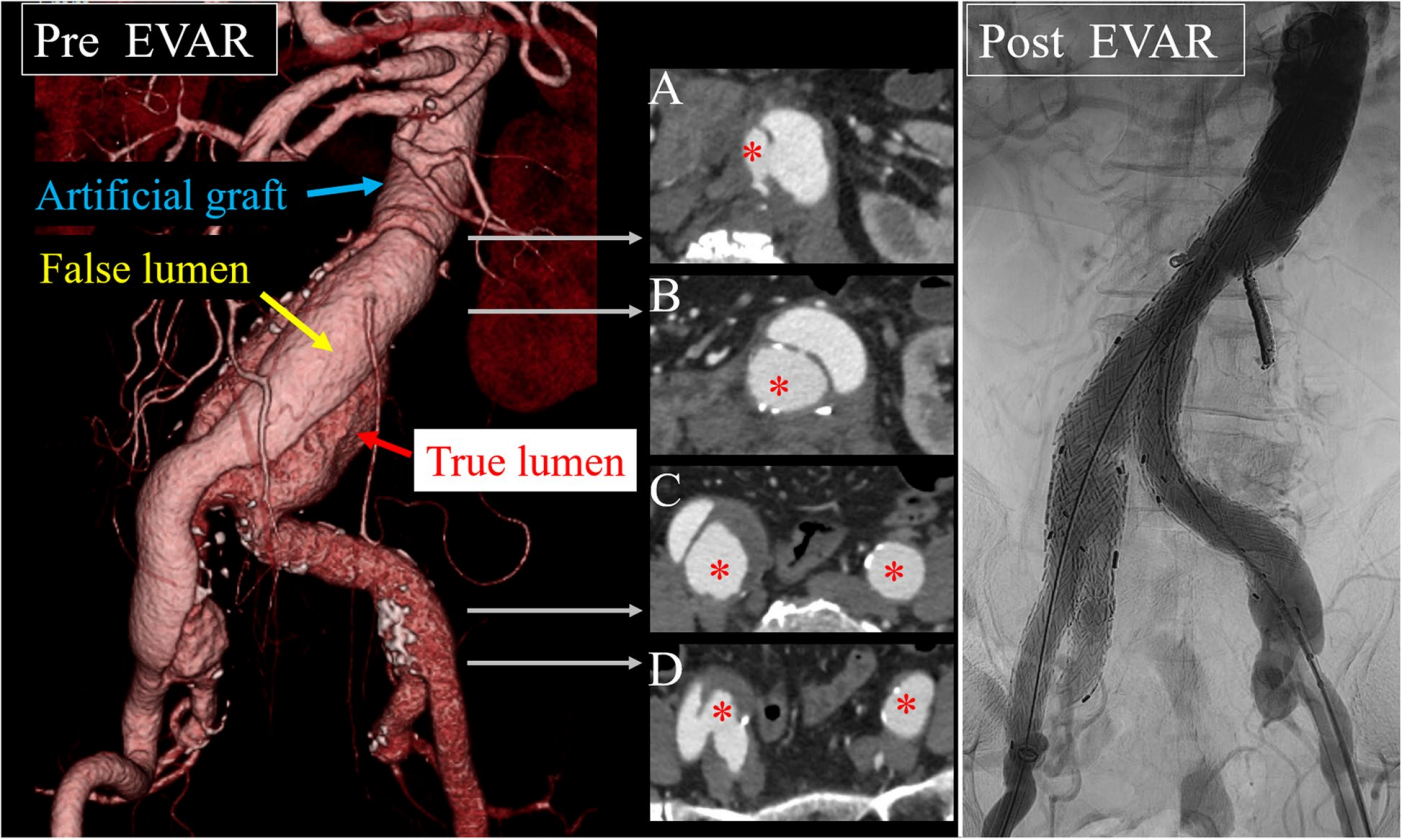
Pre-EVAR-contrast-enhanced CT and post-EVAR-angiography. **A** Distal surgical graft with a double-barrel anastomosis below the renal artery and a large entry at the same site. **B** The inferior mesenteric artery branches from the false lumen. **C**, **D** Re-entry at the right iliac artery bifurcation and termination of the dissection. *: indicate true lumen. Contrast immediately after EVAR. Angiography reveals no obvious endoleak. EVAR, endovascular aortic repair; CT, computed tomography

A 70-year-old man underwent conservative treatment for acute type B aortic dissection 5 years ago. He was referred to our hospital for enlargement of thoracoabdominal aortic and abdominal aortic and right common iliac artery aneurysms. Contrast-enhanced computed tomography (CT) revealed type B aortic dissection and a thoracoabdominal aneurysm with a maximum short diameter of 55 mm, an abdominal aortic aneurysm with a maximum short diameter of 55 mm, and a right common iliac artery aneurysm with a maximum short diameter of 40 mm. The true lumen of the thoracoabdominal aorta was narrow (Fig. 1). Since the patient had a concomitant right common iliac artery aneurysm and the reconstruction of the right iliac artery would have been difficult in a one-stage operation, a two-stage surgical strategy of thoracoabdominal aortic graft replacement and abdominal aortic graft replacement, separately, was considered. Thoracoabdominal aortic graft replacement from the proximal descending aorta to the infrarenal abdominal aorta was performed using a triplex 24-mm prosthesis (Terumo Aortic, Inchinnan, UK) without any problem. Contrast-enhanced CT after thoracoabdominal aortic graft replacement revealed the distal surgical graft had a double-barrel anastomosis below the renal artery. A re-entry tear at the right iliac artery bifurcation terminated the dissection. The true lumen was dorsal, with the right third lumbar artery located near the double-barrel anastomosis. The false lumen was ventral, with the inferior mesenteric artery branching from the false lumen (Fig. 2 Pre EVAR).

We recommended abdominal aortic graft replacement; however, he preferred EVAR over abdominal aortic graft replacement due to the prolonged rehabilitation time following his first thoracoabdominal replacement operation. Therefore, we considered the possibility of EVAR. The first problem was the necessary embolization of the internal iliac artery to treat common iliac artery aneurysms in conventional EVAR. In this case, he underwent thoracoabdominal aortic graft replacement, and the right internal iliac artery may be a source of collateral blood flow to the spinal cord, presumably increasing the risk of postoperative paraplegia. The second problem was whether the stent graft could control blood flow into the false lumen. The first problem could be solved using IBE, preserving the progressive blood flow in the internal iliac artery. The inferior mesenteric artery is the only branch originating from the false lumen. The second problem could be solved by embolizing the artery. Thus, we considered EVAR feasible.

The inferior mesenteric artery was embolized under local anesthesia the day before surgery. As the third lumbar artery was close to the entry, there was a risk of residual blood flow into the false lumen, and embolization was simultaneously performed. EVAR was performed under general anesthesia with Gore Excluder conformable (W. L. Gore and Associates, Newark, USA). A pigtail catheter was inserted from the right side into the abdominal aorta. Angiography confirmed the true lumen due to a re-entry tear in the right iliac artery. The Iliac Branch Component (IBC; CEB231410A) was placed in the right common iliac artery. An Internal Iliac Component (IIC; HGB161407A) was deployed from the IBC to the right internal iliac artery, and the re-entry tear at the right iliac artery bifurcation was closed. A Gore Excluder conformable main body (CXT321414) was inserted from the left side, and the main body was deployed from the thoracoabdominal prosthetic graft over the anastomosis of the true lumen. The contralateral leg (PLC271000J) was bridged to the right leg of the main body and IBC. The left leg of the main body and the contralateral leg (PLC231000J) were deployed. Following posterior dilatation of each part, angiography revealed no obvious endoleak, and the surgical procedure was complete (Fig. 2 Post EVAR). The operative time was 1 h 34 min, and blood loss was 20 ml.

**Fig. 3:**
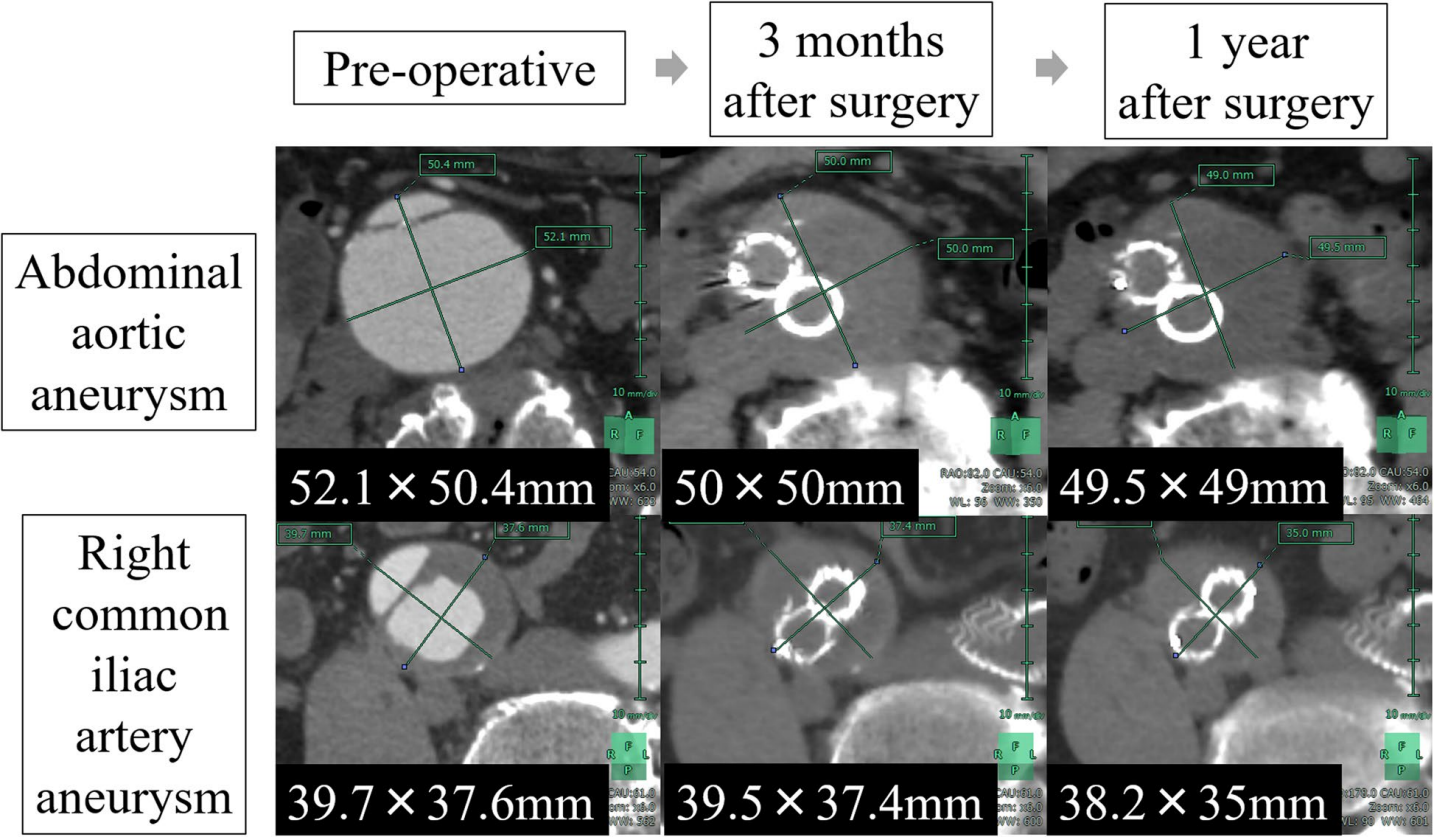
Pre- and postoperative CT images. Changes in diameter of abdominal aortic and right common iliac artery aneurysms. CT showing aneurysm shrinkage. CT, computed tomography

The patient was discharged without complications 9 days postoperatively. One-year follow-up showed that the abdominal aortic aneurysm decreased from 52.1 to 49.5 mm in maximum diameter, and the right common iliac artery aneurysm decreased from 39.7 to 38.2 mm in maximum diameter on CT; the patient was doing well (Fig. 3).

## Discussion and conclusions

Ischemic spinal cord injury in the perioperative aortic surgery is caused by reduced blood flow due to occlusion of the nutrient arteries and embolism. It is impossible to preserve antegrade blood flow in all intercostal and lumbar arteries to perform aortic replacement and stent grafting; thus, collateral vessels are important. The left subclavian [3] and lateral sacral [4] arteries, both branches of the internal iliac artery [5], are important for spinal blood flow. In this case, the internal iliac artery may be a collateral blood passage to the spinal cord after thoracoabdominal aortic graft replacement. Using IBE, EVAR after thoracoabdominal aortic graft replacement could safely be performed without embolizing the internal iliac artery.

Open abdominal aortic repair is preferable when multiple connections are challenging to manage with EVAR. In cases where thoracoabdominal aortic graft replacement has been performed, there are frequently a few connections with false lumen, making EVAR a viable option. Hybrid surgery, which combines open thoracoabdominal aortic repair with EVAR, offers a more minimally invasive approach.

The key difference between this case and a standard EVAR is that the right common iliac artery is dissected. If the re-entry is in the common iliac artery, the catheter can easily enter the false lumen, necessitating confirmation that it is in the true lumen. There are established criteria for common iliac artery diameter to ensure proper IBC deployment. However, in this case, the true lumen diameter was within the range of appropriate IBE use. When performing EVAR with IBE, 12 Fr sheath is introduced over the through-wire via femoral access contralateral to the IBE treatment side, which may place more strain on the intima than standard EVAR.

There were two reports of EVAR for aortic dissection. One involved a chronic case, 14 years after the onset of dissection [1]. The other case was acute; however, EVAR was performed after 1 month of medical treatment [2]. In this case, 5 years have passed since the onset of dissection, leading us to consider the intima stable. However, applying this approach to an acute dissection would be hazardous.

Despite anatomic limitations, EVAR can be safely performed after thoracoabdominal aortic graft replacement for chronic aortic dissection using IBE. One year postoperatively, CT showed no enlargement of the aneurysms. Currently, the course is comparable to a standard EVAR. However, only a few similar cases have been reported, and careful follow-up should be continued to ensure that the aneurysm does not enlarge.

## Data Availability

Not applicable.

## Abbreviations

IBE: Iliac branch endoprosthesis
EVAR: Endovascular aortic repair
CT: Computed tomography
TEVAR: Thoracic endovascular aortic repair
